# Exploring the “Black Box” of Recommendation Generation in Local Health Care Incident Investigations: A Scoping Review

**DOI:** 10.1097/PTS.0000000000001164

**Published:** 2023-09-15

**Authors:** William Lea, Rebecca Lawton, Charles Vincent, Jane O’Hara

**Affiliations:** From the ∗ York & Scarborough Teaching Hospital NHS Foundation Trust, University of Leeds, Leeds; † Learning & Research Centre, York Hospital, York; ‡ Psychology of Healthcare, and NIHR Yorkshire and Humber Patient Safety Translational Research Centre, University of Leeds, Leeds; § Psychology, University of Oxford, Oxford; ∥ Healthcare Quality and Safety; ¶ Yorkshire Quality & Safety Research Group, School of Healthcare, Baines Wing, University of Leeds, Leeds, United Kingdom.

**Keywords:** serious incident, incident investigation, coot cause analysis, recommendations, adverse event, hospital, patient safety

## Abstract

**Background:**

Incident investigation remains a cornerstone of patient safety management and improvement, with recommendations meant to drive action and improvement. There is little empirical evidence about how—in real-world hospital settings—recommendations are generated or judged for effectiveness.

**Objectives:**

Our research questions, concerning internal hospital investigations, were as follows: (1) What approaches to incident investigation are used before the generation of recommendations? (2) What are the processes for generating recommendations after a patient safety incident investigation? (3) What are the number and types of recommendations proposed? (4) What criteria are used, by hospitals or study authors, to assess the quality or strength of recommendations made?

**Methods:**

Following PRISMA-ScR guidelines, we conducted a scoping review. Studies were included if they reported data from investigations undertaken and recommendations generated within hospitals. Review questions were answered with content analysis, and extracted recommendations were categorized and counted.

**Results:**

Eleven studies met the inclusion criteria. Root cause analysis was the dominant investigation approach, but methods for recommendation generation were unclear. A total of 4579 recommendations were extracted, largely focusing on individuals’ behavior rather than addressing deficiencies in systems (<7% classified as strong). Included studies reported recommendation effectiveness as judged against predefined “action” hierarchies or by incident recurrence, which was not comprehensively reported.

**Conclusions:**

Despite the ubiquity of incident investigation, there is a surprising lack of evidence concerning how recommendation generation is or should be undertaken. Little evidence is presented to show that investigations or recommendations result in improved care quality or safety. We contend that, although incident investigations remain foundational to patient safety, more enquiry is needed about how this important work is actually achieved and whether it can contribute to improving quality of care.

## The “Black Box” of Recommendation Generation

Since the inception of the patient safety “movement,” efforts to improve patient safety within hospitals have relied heavily on the retrospective investigation of adverse events.^1^ Retrospective incident investigations as a mechanism for safety improvement are founded on an interpretation of safety theory, which proposes that errors are multifactorial in nature and that identifying and addressing organizational latent failures through investigation and recommendations will reduce future recurrence.^2,3^

In recent years, the generation of recommendations, after incident investigations, has come under increasing academic scrutiny.^4–7^ This interest has occurred in parallel with the establishment of national-level independent investigatory bodies (e.g., HSIB in the UK, Norwegian Healthcare Investigation Board in Norway),^8,9^ and in the UK, an ever increasing number of public inquiries and the ever expanding set of associated recommendations (e.g., Kirkup,^10^ Ockenden,^11^ Infected Blood Inquiries^12^). Therefore, exploring the act of recommendation generation is of increasing relevance as the number of recommendations across both local and national level investigation activity grows exponentially.

Although there are a plethora of aims and processes for investigations, a consistent feature is the production of recommendations. Despite 3 decades of incident investigation activity in health care,^13^ few studies have critically examined the process.^5,14^ In addition to the lack of empirical work examining recommendation generation, there is a lack of practical guidance, on the generation of recommendations.^6^ One systematic review used a modified version of the National Institute for Occupational Safety and Health hierarchy of risk controls to categorize the recommendations from included studies,^5,15^ concluding that 80% of recommendations were “weak,” that is, unlikely to result in significant improvements in safety or risk reduction. Furthermore, Hibbert and colleagues^16^ undertook a retrospective study, following investigations within an Australian regional health system. The study used and modified the U.S. Department of Veteran Affairs action hierarchy (AH) to categorize recommendations as strong, medium, or weak and concluded that only a small number of recommendations were strong and the most common types of recommendations involved reviewing or enhancing policies/guidelines/documentation as well as training and education.^16^ It is important to note that these issues extend beyond health care. Indeed evidence suggests that a lack of guidance and a plethora of other sociotechnical factors impede the generation, implementation, and evaluation of recommendations across safety investigations in contexts such as rail, maritime, and nuclear.^6,17^

## Recommendation Generation Within Local Health Care Investigations

Despite the centrality of incident investigation and recommendation generation within patient safety policy globally, there is a surprising lack of understanding about what actually happens in local health care settings with respect to this important activity. In particular, there is a lack of empirical focus and consensus about recommendation generation by people conducting investigations at a local health care organization level.^4,13^ This review therefore aims to examine the extant empirical knowledge about this issue. We have focused on hospital settings rather than primary/community care because of the fundamentally different ways in which care is delivered and case mix,^18^ as well as the relatively lower level of incident reporting and relevant published literature in primary care.^18–20^

## Scoping Review Aims

The purpose of this review was to consider the following questions:

1. What approaches to incident investigation are used before the generation of recommendations?
2. What are the processes for generating recommendations after a patient safety incident investigation?
3. What are the number and types of recommendations proposed?
4. What criteria are used, by hospitals or study authors, to assess the quality or strength of recommendations made?

## METHODS

We conducted a scoping review, following the preferred reporting items for systematic reviews and meta-analyses extension for scoping reviews guidance.^21^

### Sources and Searches

Searches were performed on February 28, 2019, and January 30, 2021, using MEDLINE, EMBASE, PsychINFO, and CINAHL. Search terms were iteratively developed to capture the key phases of incident investigation including terms for the incident, investigation, and subsequent recommendations (see Appendix 1 for search terms, http://links.lww.com/JPS/A565). Searches were restricted to English language and studies published since 1999, when the Institute of Medicines’ seminal report, *To Err Is Human*, was published,^22^ prompting greater focus on patient safety.

### Study Selection

The aim of this review was to examine the routine investigation and recommendation generation processes that occur in hospitals.

Studies were included if they reported on a series of incidents occurring in the hospital, which were chosen for investigation by hospital-based staff, who also generated subsequent recommendations. Studies reporting on incidents from any clinical context or level of harm were included.

Studies were excluded if they reported data from the following:

1. Community, primary care, or primarily mental health care
2. Investigations/recommendations carried out or proposed outside of a hospital, for instance, by an external research team or regional organization
3. Investigations primarily carried out for the purposes of research
4. Not published/peer-reviewed (e.g., conference papers)

**FIGURE 1:**
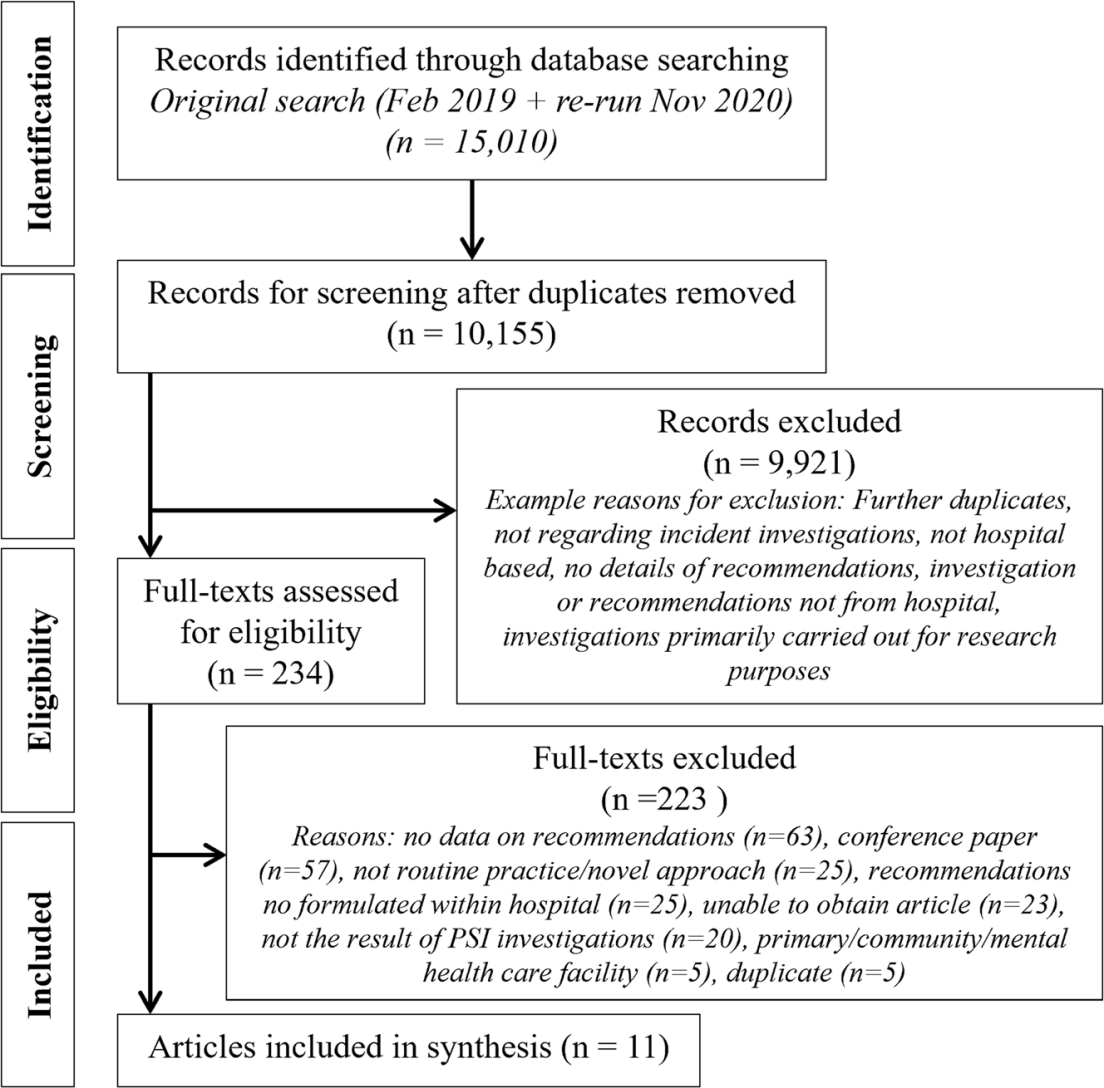
PRISMA-ScR flow diagram for study selection.

Searches yielded 15,010 articles. The article title and abstracts were reviewed by W.L. Random samples of 5% (n = 720) were screened independently by both J.O.H. and R.L. to check congruence. A total of 246 articles were selected for full-text review. Full-text screening was undertaken by W.L., with 10% independently screened by each of J.O.H. and R.L. (n = 20). Any discrepancies were discussed and resolved between authors. Eleven articles met the inclusion/exclusion criteria (all agreed with W.L., J.O.H., and R.L.) and contributed to the review (Fig. 1). Regular meetings with the other author (C.V.) allowed discussion of article eligibility.

### Data Extraction and Quality Assessment

The purpose of the review was to examine the nature of recommendations proposed within hospitals, which was not the primary aim of all the included studies, but those included did contain empirical data on recommendations.

We assessed study quality using the Quality Assessment for Diverse Studies (QuADS) tool.^23^ This tool is a well-cited approach to assessing the quality of methodologically heterogeneous studies, which demonstrates reliability and validity.^23,24^ After discussion of the application of the tool and relevance of quality scoring by all the authors, W.L. reviewed and scored all included articles. A random sample (n = 4 [36%]) of studies were independently reviewed and scored by J.O.H. and R.L., with disagreements resolved with discussion.

### Data Synthesis and Analysis

To address research questions 1, 2, and 4, we undertook content analysis of the included studies using 4 stages; decontextualization, recontextualization, categorization, and compilation.^25^ First, authors read and made themselves familiar with the included studies before extracting “meaning units” of text relevant to answering the aims of the review (decontextualization). After extraction of meaning units, the remaining article text was checked for further relevant content (recontextualization). Next the extracted meaning units were split into specific areas relevant to each research question; the word count was reduced without losing the meaning/content (categorization). The research questions were answered by condensing the extracted text using the original study terms and language, as well as providing numerical counts of how often content was reported across the studies.

To address research question 3, we used the AH, proposed by the U.S. Veteran Affairs National Center for Patient Safety to categorize the recommendations extracted from included studies.^16,26–29^ Recommendations from the included studies were discussed by all the authors across 2 meetings and assigned to the core categories of the AH, then counted, to report frequency. If, after discussion, it was felt that a recommendation or category of recommendations did not fit into one of the AH categories, a new category was created and agreed.

## RESULTS

The characteristics of included studies (n = 11) are summarized in Table 1. Included studies contained 4680 recommendations from 2818 investigations carried out across 171 hospitals.

**TABLE 1:** Summary of Included Studies

Author (Year)	Reference No.	Country	Clinical Context	No. hospitals	No. Incidents Analyzed	Types of Incident(s)/Incident(s) Relating to	Levels of Harm of Investigated Incident(s)	No. recommendations	How Are Recommendations Categorized?
Corwin et al (2017)	^30^	United States	Intensive care	47	70	Delay in care; medication; medical procedure; equipment failure; removal of lines, catheters, tubes, drains; transfusion, elopement; discharge; suicide attempt; fall; airway/ventilation	Incidents causing harm only	276	Categories developed by authors based on included investigations recommendations
Figueiredo et al (2018)	^31^	Brazil	Tertiary general hospital	1	1316	Drug supply chain; fall; pressure ulcer; other skin lesions; surgical procedure (relation with laterality); transfusion process; unplanned withdrawal of catheter, drain, tube or catheter; Identification of patient; loss of sample; bruise; extravasation; delay in exam/procedure completion; prolonged fasting; failure to release the technical report; evasion; technical or equipment/material handling failure; failure to identify material/instruments; related to childbirth; nutritional therapy; health care related infection; failure during technique, procedure or transportation; death and others	No harm and harm incidents included	1326	Categories developed by authors based on included investigations recommendations
Hamilton et al (2019)	^27^	Australia	Hospitals/pediatric	16	42	Delayed diagnosis; delayed recognition or response to a deteriorating patient; procedural adverse event; patient identification or procedure mismatching; medication adverse event; delayed definitive treatment; unexpected death/event after hospital presentation or admission; testicular torsion delayed diagnosis or management	Incidents causing harm only	150	U.S. Department of Veteran Affairs’ criteria, or AH (VA AH)
Hibbert et al (2018)	^16^	Australia	Hospitals in region	36	227	Clinical process/procedure; falls; behaviors; problems with diagnosis; problems with procedures or interventions; wrong patient/body part; inpatient suicide; retained instruments/other; gas embolism; ABO incompatible blood transfusion; medication error; maternal death; wrong infant discharged	Incidents causing harm only	1137	U.S. Department of Veteran Affairs’ criteria, or AH (VA AH)
Irwin et al (2011)	^32^	United Kingdom	Hospitals in region/pharmacy	23	573	Dispensing of medications	No harm and harm incidents included	251	Categories developed by authors based on included investigations recommendations
Kellogg et al (2017)	^33^	United States	Academic Medical Centre	1	302	Procedure complication; cardiopulmonary arrest; neurological deficit; retained foreign body; pulmonary/arterial embolus; birth complication; medication administration error; incorrect procedure/study; sepsis; wrong-site surgery/procedure; devastating illness; myocardial infarction; hemorrhage/hematoma; arrhythmia; unknown cause of death; adverse medication event; compartment syndrome; fall, inpatient; event proximate to discharge; self-harm; electrolyte disturbance; assault, inpatient; bowel perforation; equipment failure; sleep apnea; ventilation complication	No harm and harm incidents included	499	Categories developed by authors based on included investigations recommendations
Kwok et al (2020)	^28^	Hong Kong	Hospitals in region	43	214	Surgery/interventional procedure involving the wrong patient or body part; retained instruments or other material after surgery/interventional procedure; ABO incompatibility blood transfusion; intravascular gas embolism resulting in death or neurological damage; death of an inpatient from suicide; maternal death or serious morbidity associated with labor or delivery; infant discharged to wrong family or infant abduction; other adverse events resulting in permanent loss of function or death; medication error which could have led to death or permanent harm; patient misidentification which could have led to death or permanent harm	No harm and harm incidents included	760	U.S. Department of Veteran Affairs’ criteria, or AH (VA AH)
Morse and Pollack (2012)	^26^	United States	Pediatric hospital	1	20	Medication event; delayed identification of clinical deterioration; equipment failure; enteral feeding via central line; breast milk event; unsterile surgical equipment; inappropriate patient behavior in the “play room”; significant tissue injury; name change patient identification; readmission event; wrong site MRI under general anesthesia	No harm and harm incidents included	78	U.S. Department of Veteran Affairs’ criteria, or AH (VA AH)
Robbins et al (2020)	^34^	United Kingdom	University hospital	1	22	No details	Incidents causing harm only	101*	Hierarchy of intervention effectiveness (people versus system focused) (Cafazzo and St-Cyr^35^)
van der Starr et al (2014)	^36^	The Netherlands	Neonatal/pediatric intensive care	1	17	Medication errors; procedural; unanticipated death, unanticipated resuscitation; nursing care	No harm and harm incidents included	84	Factors influencing clinical practice devised by Woloshynowych et al^3^
Zeng et al (2016)	^37^	United States	Hospital	1	15	Reported most common: communication failure between team members; tasks to automate for dosimetry; simulation issues; IT system failures; scheduling issues	No harm and harm incidents included	18	Categories developed by authors based on included investigations recommendations
Total				171	2818			4579	

*Lacked detail to enable categorization and therefore not included in Table 3.

### Country of Origin

Included studies were conducted in the United States (n = 4), the United Kingdom (n = 2), and Australia (n = 2), with one each from the Netherlands, Brazil, and Hong Kong.

### Clinical Context and Incident Harm

Studies reported data from across all clinical specialties (n = 6), pharmacy/medication (n = 1), anesthesia and intensive care (n = 2), and pediatric care (n = 2). Incidents reported within studies varied in their type (e.g., delay in care, fall, dispensing of medication) and resulting harm (see Table 1 for more detail).

### Quality Assessment

The included studies demonstrated an average QuADS score of 56% (range, 26%–69%) Five of 11 studies lacked theoretical underpinning such as the discussion of an accident causation model. Half of the studies did not report, in sufficient detail, the justification of sampling or selection of data collection tools. Six studies had no evidence that research stakeholders had been involved in their planning or conduct. Four studies had limited or no discussion of their strengths or limitations. No studies were excluded based on quality.

### RQ1) Approaches to Incident Investigation Used Before the Generation of Recommendations

Nine studies reported using root cause analysis (RCA),^16,26–28,30,33,34,36,37^ 3 used both RCA and the London Protocol,^16,36,37^ and the remaining 2 used no specific tool or method.^31,32^ Four studies reported that a team of 2 to 8 staff (physicians, nurses, and managers) undertook the investigation,^28,33,36,37^ and 2 reported specific investigator training.^34,36^ The remaining studies did not provide these details.

As part of the investigation process, 3 studies reported interviewing staff,^33,36,37^ one of which specified that incidents were reconstructed from a median of 6 interviews (n = 3–15).^36^ One study reported that parents of children involved in incidents were interviewed “if felt to be useful,” and this occurred in 2 of 17 incidents.^36^

Four studies reported on the time spent undertaking investigations. This was highly variable, ranging from 3 to 90 hours.^26,34,36,37^ Three studies reported that investigations should be completed within a set period of time, ranging from 30 to 60 days,^28,30,33^ although they did not specify if this was from when the incident occurred or was reported, or the decision to investigate was made.

### RQ2) The Processes for Generating Recommendations After A Patient Safety Incident Investigation

None of the included studies reported using specific tools or methods for recommendation generation. One article reported that staff and parents were invited to suggest recommendations, whereas none of the remainder reported this kind of stakeholder involvement.^36^ Eight studies proposed that recommendations should prevent incident recurrence^16,27,28,30,33,34,36,37^ and eliminate, mitigate, or reduce a risk, hazard, or “root causes.”^28,30,33,34^ No purpose or aim for recommendations was stated in the remaining 3 studies.

### RQ3) The Number and Types of Recommendations Proposed

A variety of terms were used to describe the recommendations generated after investigations. We present these terms in Table 2, but because the terms were not clearly defined within the studies, we were not able to determine differences or similarities and have therefore reported them as written. A total of 4579 recommendations were extracted from 10 included studies (Table 3), with an average of 3.7 (1–5) per investigation. Recommendations were not extracted from the 11th included study because of insufficient detail to enable categorization.^34^ Six studies assigned recommendations to predetermined categories based on (i) the U.S. Department of Veteran Affairs’ criteria or AH,^16,26–28^ (ii) factors influencing clinical practice devised by Woloshynowych et al,^3,36^ or (iii) the “hierarchy of intervention effectiveness” (people versus system focused).^34^ The remaining 5 studies developed their own categories based on analysis of their included recommendations.^30–33,37^

**TABLE 2:** Terms Used to Describe the Recommendations After Investigations

	Frequency	Study Reference
Recommendations	5	Hibbert et al,^16^ Hamilton et al,^27^ Kwok et al,^28^ Corwin et al,^30^ van der Starre et al^36^
Action(s)	5	Kwok et al,^28^ Corwin et al,^30^ Figueiredo et al,^31^ Kellogg et al,^33^ Robbins et al^34^
Action plan(s)	2	Morse and Pollack,^26^ Zeng et al^37^
Corrective actions/action plans	2	Morse and Pollack,^26^ Robbins et al^34^
Solutions	2	Kellogg et al,^33^ Robbins et al^34^
Process improvements	1	Zeng et al^37^
Interventions	1	Irwin et al^32^
Risk reduction strategies/measures	1	Morse and Pollack^26^
Preventative measures	1	van der Starre et al^36^
Recommended actions	1	Corwin et al^30^
Managerial responses	1	Irwin et al^32^
Error management strategies	1	Irwin et al^32^
Risk controls	1	van der Starre et al^36^
Process improvement projects	1	Zeng et al^37^

**TABLE 3:** Recommendations Extracted From Included Studies

AH Strength of Recommendations	Recommendation Category No.	Recommendation Categories	n	% Within AH	% All Recommendations
Strong	1	Standardize on equipment or process	66	2.0	1.4
	2	Architectural/physical plant changes	60	1.8	1.3
	3	Tangible involvement by leadership	44	1.3	1.0
	4	New devices with usability testing	23	0.7	0.5
	5	Engineering control (forcing function)	16	0.5	0.3
	6	Simplify process	14	0.4	0.3
Total strong			223	6.8	4.9
Medium	7	Adjust or improve a policy or guideline	306	9.4	6.7
	8	Enhanced documentation or communication	170	5.2	3.7
	9	Audit undertaken	149	4.6	3.3
	10	Checklist or cognitive aids	90	2.8	2.0
	11	Software enhancements or modifications	69	2.1	1.5
	12	Analyze/inspect/review use or appropriateness of equipment	37	1.1	0.8
	13	Review rostering/appropriateness of staff mix	32	1.0	0.7
	14	Increase in staffing/decrease in workload	17	0.5	0.4
	15	Standardized communication tools	12	0.4	0.3
	16	Education using simulation-based training, with periodic refresher sessions and observation	13	0.4	0.3
	17	Redundancy	9	0.3	0.2
	18	Eliminate/reduce distractions	9	0.3	0.2
	19	New [clinical] team	5	0.2	0.1
	20	Eliminate look- and sound-alikes	1	0.0	0.0
Total medium			919	28.2	20.1
Weak	21	Training	1257	38.5	27.5
	22	New procedure/memorandum/policy	676	20.7	14.8
	23	Meeting to discuss event/staff made aware of event	105	3.2	2.3
	24	Staff asked to provide written reflective statement or staff informed/notified/warned	30	0.9	0.7
	25	Double checks	27	0.8	0.6
	26	Warnings	25	0.8	0.5
Total weak			2120	65.0	46.3
		Total categorized within AH	3262	100.0	
New categories	27	Vague/unclear	656		14.3
	28	Change of process/routine	500		10.9
	29	Additional study/analysis	121		2.6
	30	Risk assessment/management/risk register	12		0.3
	31	Supervision	12		0.3
	32	Involvement of external organization (external investigating or contacted as part of investigation)	10		0.2
	33	New staff role	1		0.0
	34	Purchase new equipment	3		0.1
	36	Adjustments to team expertise/make-up	2		0.0
Total recommendations within new categories			1317		28.8
Total recommendations extracted			4579		100.0

Education or training represented the most common recommendation (27.2% [n = 1257]), followed by new procedure/memorandum/policy (15% [n = 676]), change of process or routine (10.7% [n = 500]), and adjustment/improvement to policy or guideline (6.7% [n = 306]). Fourteen percent of the extracted recommendations were too vague or unclear to categorize. Table 3 shows the full breakdown of recommendations by category. Recommendation categories 1 to 26, in Table 3, are from the AH,^16,26–28^ and categories 27 to 36 are those proposed by the study authors. Six hundred fifty-six recommendations were categorized as “vague/unclear” either by the authors of the included studies or authors of this review during analysis. Examples of vague/unclear recommendations included “Medication incident action plan implemented” (n = 3)^32^; “policy, procedure and process actions” (n = 5)^30^; and “provide counseling” (n = 280).^31^

### RQ4) Criteria Used to Assess the Quality or Strength of Recommendations Made

Two of 11 articles reported that the original internal hospital investigations made judgments of recommendation “quality” or “strength.”^30,37^ One study reported that the hospital prospectively tagged incidents to identify trends and therefore monitor for process improvements, although it did not report any data in relation to this.^37^ Another study reported that implemented action (n = 277) effectiveness was rated by local managers as “much better” (47.4%), “better” (37.0%), “same”(7.4%), “worse” (0%), or not reported or measured (8.2%).^30^ Although none the studies provided comprehensive data on incident recurrence, one study reported that similar incidents did reoccur despite multiple investigations.^33^

Included studies, in secondary analysis, used a range of terms or phrases to “judge” recommendations as follows.

- Effectiveness (Hibbert et al,^16^ Kwok et al,^28^ Corwin et al,^30^ Figueiredo et al,^31^ Kellogg et al,^33^ van der Starre et al,^36^ Robbins et al^34^)
- Strength (Hibbert et al,^16^ Morse and Pollack,^26^ Hamilton et al,^27^ Kwok et al,^28^ Kellogg et al^33^)
- Whether implemented (Morse and Pollackm^26^ Hamilton et al,^27^ Corwin et al,^30^ Kellogg et al,^33^ van der Starre et al^36^)
- Aimed at system level improvements or modifying processes (Morse and Pollack,^26^ Kwok et al,^28^ Kellogg et al^33^)
- Likelihood they would prevent incident recurrence (Morse and Pollack,^26^ Kellogg et al,^33^ van der Starre et al^36^)
- Quality (Morse and Pollack,^26^ Robbins et al^34^)
- Sustainability (Hibbert et al,^16^ Kellogg et al^33^)
- Efficacy (Hamilton et al^27^)
- Innovation (Robbins et al^34^)
- Level of impact (Morse and Pollack^26^)

In 8 of 11 studies, authors discussed their approach to judging recommendations.^16,26–28,30,33,34,36^ Four studies judged recommendations as strong, intermediate, or weak based on the AH with some variations in the category descriptions and/or addition of further categories.^16,26–28^ One study referenced a “Model of Sustainability and Effectiveness in RCA Solutions,”^33,38^ WHEREAS another reported effectiveness of recommendations according to the “Hierarchy of Intervention Effectiveness,” which proposes that “system-focused changes have greater impact.”^34,35^ One article commented on recommendation likelihood of preventing incident recurrence,^36^ based on a classification of recommendation strength (weak, medium, strong) proposed by the New South Wales Root Cause Analysis Review Committee.^39^

## DISCUSSION

To the author’s knowledge, this review represents the first review of the extant empirical evidence for the practice of generating recommendations in hospitals, specifically examining how and what recommendations were generated, as well as the way in which their effectiveness was judged. This process is central to the efforts to improve patient safety and health care quality globally. Our review highlights the paradoxical situation that, despite the ubiquity of recommendation generation, very little is known about it in practice. Our findings suggest that, although RCA dominates as the approach to investigation, there are no specific tools or approaches used to generate recommendations. Recommendations focus on training or adding or improving policies. In other words, recommendations largely focus on staff knowledge and skills. There is a lack of agreement in the literature on how effectiveness of recommendations should be judged, meaning that there is very little understanding of what makes a “good” recommendation. These findings raise some important issues, which we will address in turn.

### Recommendation Generation Is Confused and Unclear

The variety of terms used to describe recommendations (Table 2) and lack of consensus for categorization suggests differences in vision and purpose at best, and confusion and disagreement at worst. Although this review provides some steer in terms of the espoused investigation techniques, the actual process of how investigation outcomes result in specific recommendations remains opaque. We found that, beyond the investigators, there are committees or teams within hospitals as well as within local or regional organizations that review investigations and their findings; although what role these groups had in selecting or modifying recommendations is unclear. Studies in the wider literature have attempted to explore this process in practice. Braithwaite et al^40^ found a number of challenges to RCA such as time constraints, lack of resources, and unwilling colleagues. Another study suggested that recommendations may actually be related to other ongoing improvement work; that is, the incident was used to support existing agendas rather than to generate new findings.^41^ Furthermore, an ethnography of investigations identified attempts by investigators to manage scrutiny and maintain reputations, and concluded that a failure to appreciate the complex organizational agendas as well as social and political influences on recommendation generation would likely hamper improvements in patient safety.^42^ Beyond health care, studies of investigations from other domains, such as nuclear and rail, have demonstrated that the design of approaches to investigation and associated manuals lack emphasis or detail on the generation and evaluation of recommendations.^6^ Another cross-domain study identified that there are a large number of cognitive, political contextual factors that influence the investigation and recommendation generation process, such as cost-benefit analysis, willingness of stakeholders to engage, or the experience or knowledge base of the investigator.^17^ Collectively, these studies suggest that the generation of recommendations is likely to be a highly complex sociopolitical process with many stages and influences.^4,40–43^ New approaches and tools for recommendation generation^44–47^ are more likely to be successful if adapted and designed relative to the unique and complex context of health care.^48,49^ Further research to understand the reality of the movement from investigation to recommendation generation is therefore important.

### Recommendations Are Classified as Weak and Lack System Focus

This review identified that less than 7% of the extracted recommendations might be considered “strong” or system-focused, such as standardizing equipment, architectural changes, or simplifying processes. Our findings provide further evidence for the continued tendency for “weaker” recommendations that focus on improving individuals’ behavior and practice, rather than the wider system deficiencies that contribute to incidents. This tendency, shown in numerous studies from across the globe,^5,50–57^ suggests explanatory reasons beyond national culture or specific differences in health care systems and is completely at odds with health care policy and safety research.^3,29,39^ Furthermore, it would suggest that, globally, health care organizations may have some way to go toward achieving a more just culture, with this focus on weaker individual-focused recommendations both reflecting this and serving to reinforce it.^2^

Root cause analysis and frameworks, used to support investigation, have themselves been identified as narrowing the view of causation^4^ or giving greater attention to causative factors relating to individuals.^58^ With a tendency for investigations to identify individual factors,^58^ it is perhaps not surprising that recommendations are targeted at the same level. Other reasons for a lack of system-level recommendations include lack of investigator training, expertise,^5^ or health care–tailored guidance,^3^ and difficulty in designing and implementing at the system level.^15,48^ This review highlights the continued predominance of RCA, despite the growing number of alternatives that might broaden investigations and identify a wider range of contributory factors and subsequent recommendations.^59–63^

### It Is Not Clear How to Judge Recommendations

Although the focus of recommendations at the weaker individual level has been widely challenged, a further compounding problem with recommendation generation is the lack of agreement on how to judge their effectiveness and what makes a “good” recommendation. The range of terms, in our included studies, such as “strength,” “quality,” “sustainability,” and “implementability,” indicates the complex nature of judging recommendations. Our review found 2 broad approaches: (i) the use of predefined hierarchies of recommendation effectiveness and (ii) assessing the effectiveness of recommendations over time.

Starting with hierarchies, this review demonstrates not only their widespread use but also variety and variation.^5,15,16,26,28,29,35,38,39,64–66^ These hierarchies, largely originating from non–health care settings,^48,67^ are used in health care with minimal empirical evidence.^38^ They generally propose that recommendations targeted at the individual level (e.g., training and reminders) are weaker than those at the system level (e.g., equipment design). Before this review, there have been challenges of the use of hierarchies to predict recommendation effectiveness,^47,48^ with arguments that recommendations should be judged on how well they align with the identified risks and context,^46^ their likelihood of effecting necessary change,^68^ or level of system targeted for change.^47^ Our review suggests that hierarchies may not yet be widely used in practice, but with the growing number of variations and lack of consensus, they have the potential to cause confusion for hospital safety teams looking to adopt evidence-based approaches. Beyond the need for empirical evaluation of these options, we suggest that future research will also need to consider the practical application of these in health care.

The second approach to judging recommendation effectiveness seems to be “post-hoc” measures, more specifically assessing what difference is made to processes and outcomes, as well as future incident occurrence. In problem solving, determining the effectiveness of solutions is a key step.^67^ There is a surprising absence of post-hoc measures reported within the included studies, with none of the included studies comprehensively reporting the rates of incidence recurrence. With “the prevention of incident recurrence” being the most commonly quoted reason for incident investigation, it is of note that these data are lacking within this review, as well as the wider literature.^4,5,43^

Beyond this review, numerous studies have indicated that incident reporting systems (key for incident identification) only detect a minority of incidents that actually occur, and this number may be even lower for incidents resulting in harm.^69–71^ Incident recurrence may be a poor marker of investigation success, if reporting remains unreliable. We contend that more research is needed to consider specifically what measures are appropriate for measuring recommendation or investigation effectiveness.

Although Reasons’ organizational accident model is central to much of health care investigation practice,^2,3^ the included studies demonstrate a lack of translation of the complexity and nuance of the original model. For instance, the recommendations largely focus on reducing error rates rather than putting in place defenses to more broadly improve system safety and quality or reduce the impact of an error if it does occur. The studies included within this review provide no evidence that carrying out investigations and generating recommendations improve the quality or safety of care. Furthermore, there seems to be little consideration of the potential negative consequences of recommendations themselves.

### Limitations

Despite the volume of incident reporting and investigation within health care, there is a relative lack of peer-reviewed research with empirical data from “real-world” hospital investigations. Relevant studies may have been excluded if there was ambiguity as to whether they reported data from usual practice within hospitals, as this was the focus of the review. Because of the lack of studies exploring the specific aims of this review, the included study’s aims were not necessarily aligned with the aims of the review, rather relevant empirical data were extracted. Many of the included studies do not report the entire investigation process in detail or the effect of recommendations, which has impacted our ability to answer some of the review questions. It was not possible to analyze recommendations at the incident level, which would have allowed us to identify the proportion of recommendations at the individual and system levels. We recognize that this would be an important area for future research. Because we have focused on internal hospital investigations, as opposed to those at a regional or national level, there is a chance that this is one reason there are less observed recommendations targeting those contributory factors or organizations external to the hospital; internal hospital investigations may be more likely to focus on what they perceive they can change.^17^ This review has focused on the generation of recommendations, but no assumption is made that “good” recommendations will necessarily improve safety. Implementation of recommendations and the challenges and barriers is another important factor to consider but was beyond the scope of this review.

## CONCLUSIONS

The aim of this review was to explore hospitals’ approaches to incident investigation, recommendation generation, the types of recommendations proposed and how their effectiveness is judged. Although RCA dominates as the approach to investigation, how recommendations are selected remains unclear. Recommendations are generally classified as weak, focusing on improving individuals’ skills, knowledge, and understanding so as to change behavior rather than addressing deficiencies in the systems in which staff work. Our review demonstrates a lack of evidence and consensus regarding how recommendations should be judged for effectiveness. We argue that greater clarity is needed in terms of the purpose of investigations and the language used to describe them. Furthermore, empirical work needs to explore and explicate how to generate appropriate recommendations, as well as how these approaches are adopted within the complex sociotechnical context of health care.

Finally, we suggest that, although incident investigations remain foundational to patient safety measurement and improvement, more enquiry is needed about their effectiveness or impact. The generation of recommendations themselves is only one step in the process. Both policy and practice will also need to engage with the growing body of literature and adopt a more evidenced-based approach to investigation and recommendation selection.

## Supplementary Material
