# Supplementary material for: Exploring the “Black Box” of Recommendation Generation in Local Health Care Incident Investigations: A Scoping Review
Source: J Patient Saf. 2023 Sep 15;19(8):553–63. doi: 10.1097/PTS.0000000000001164 (PMC10662609; doi:10.1097/PTS.0000000000001164)
Supplement: Supplementary file 1 [file jps-19-553-s001.docx]

**Exploring the ‘black box’ of recommendation generation in local healthcare incident investigations: A scoping review**

Dr William Lea BM BS [CORRESPONDING AUTHOR]

York & Scarborough Teaching Hospital NHS Foundation Trust

University of Leeds

Address: Learning & Research Centre, York Hospital, Wigginton Road, York, UK, YO30 8HE

Phone: 01904 721039

Email: william.lea1@nhs.net

Professor Rebecca Lawton PhD

Professor, Psychology of Healthcare, University of Leeds

Director, NIHR Yorkshire and Humber Patient Safety Translational Research Centre

Address: University of Leeds, Leeds, UK, LS2 9JT

Phone: 0113 3435715

Emails: R.J.Lawton@leeds.ac.uk

Professor Charles Vincent PhD

Professor of Psychology, University of Oxford

Address: Anna Watts Building, Radcliffe Observatory Quarter, Woodstock Road, Oxford, OX2 6GG

Phone: +44 (0)1865 271444

Email: charles.vincent@psy.ox.ac.uk

Professor Jane O’Hara PhD

Professor of Healthcare Quality and Safety, University of Leeds

Deputy Director - Yorkshire Quality & Safety Research Group

Address: School of Healthcare, Baines Wing, University of Leeds, Leeds, UK, LS2 9JT

Phone:

Email: J.O'Hara@leeds.ac.uk

All authors declare that they have no conflicts of interest.

**Exploring the ‘black box’ of recommendation generation in local healthcare incident investigations: A scoping review**

**Abstract**

**Background**: Incident investigation remains a cornerstone of patient safety management and improvement, with recommendations meant to drive action and improvement. There is little empirical evidence about how – in real world hospital settings – recommendations are generated or judged for effectiveness.

**Objectives**: Our research questions, concerning internal hospital investigations, were: 1) What approaches to incident investigation are used prior to the generation of recommendations?; 2) What are the processes for generating recommendations following a patient safety incident investigation?; 3) What are the number and types of recommendations proposed?; 4) What criteria are used, by hospitals or study authors, to assess the quality or strength of recommendations made?;

**Methods**: Following PRISMA-ScR guidelines we conducted a scoping review. Studies were included if they reported data from investigations undertaken, and recommendations generated within hospitals. Review questions were answered with content analysis and extracted recommendations were categorised and counted.

**Results**: Eleven studies met the inclusion criteria. Root Cause Analysis was the dominant investigation approach, but methods for recommendation generation were unclear. A total of 4579 recommendations were extracted, largely focusing on individuals’ behaviour rather than addressing deficiencies in systems (less than 7% classified as strong). Included studies reported recommendation effectiveness as judged against pre-defined ‘action’ hierarchies or by incident recurrence, which was not comprehensively reported.

**Conclusion**: Despite the ubiquity of incident investigation, there is a surprising lack of evidence concerning how recommendation generation is or should be undertaken. Little evidence is presented to show that investigations or recommendations result in improved care quality or safety. We contend that whilst incident investigations remain foundational to patient safety more enquiry is needed about how this important work is actually achieved and whether it can contribute to improving quality of care.

**Introduction**

*The ‘black-box’ of recommendation generation*

Since the inception of the patient safety ‘movement’, efforts to improve patient safety within hospitals have relied heavily on the retrospective investigation of adverse events.[1] Retrospective incident investigations as a mechanism for safety improvement are founded on an interpretation of safety theory which proposes that errors are multifactorial in nature, and that identifying and addressing organisational latent failures through investigation and recommendations will reduce future recurrence.[2,3]

In recent years, the generation of recommendations, following incident investigations, has come under increasing academic scrutiny.[4-7] This interest has occurred in parallel with the establishment of national level independent investigatory bodies (e.g. HSIB in the UK, Norwegian Healthcare Investigation Board in Norway),[8,9] and in the UK, an ever increasing number of public inquiries and the ever expanding set of associated recommendations (e.g. Kirkup, Ockenden, Infected Blood Inquiries).[10-12] Therefore, exploring the act of recommendation generation is of increasing relevance as the number of recommendations across both local and national level investigation activity grows exponentially.

Whilst there are a plethora of aims and processes for investigations, a consistent feature is the production of recommendations. Despite three decades of incident investigation activity in healthcare,[13] few studies have critically examined the process.[5,14] In addition to the lack of empirical work examining recommendation generation, there is a lack of practical guidance, on the generation of recommendations.[6] One systematic review used a modified version of the National Institute for Occupational Safety and Health (NIOSH) hierarchy of risk controls to categorise the recommendations from included studies,[5,15] concluding that 80% of recommendations were ‘weak’, i.e. unlikely to result in significant improvements in safety or risk reduction. Further, Hibbert and colleagues undertook a retrospective study, following investigations within an Australian regional health system.[16] The study used and modified the US department of Veteran Affairs action hierarchy to categorise recommendations as strong, medium or weak and concluded only a small number of recommendations were strong and the most common types of recommendations involved reviewing or enhancing policies/guidelines/documentation as well as training and education.[16] It is important to note that these issues extend beyond healthcare. Indeed evidence suggests that a lack of guidance and a plethora of other socio-technical factors impede the generation, implementation and evaluation of recommendations across safety investigations in contexts such as rail, maritime, and nuclear.[6,17]

*Recommendation generation within local healthcare investigations*

Despite the centrality of incident investigation and recommendation generation within patient safety policy globally, there is a surprising lack of understanding about what actually happens in local healthcare settings with respect to this important activity. In particular, there is a lack of empirical focus and consensus about recommendation generation by people conducting investigations at a local healthcare organisation level.[4,13] This review therefore aims to examine the extant empirical knowledge about this issue. We have focused on hospital settings rather than primary/community care due to the fundamentally different ways in which care is delivered and case mix,[18] as well as the relatively lower level of incident reporting and relevant published literature in primary care.[18-20]

**Scoping review aims**

The purpose of this review was to consider the following questions:

1. What approaches to incident investigation are used prior to the generation of recommendations?
2. What are the processes for generating recommendations following a patient safety incident investigation?
3. What are the number and types of recommendations proposed?
4. What criteria are used, by hospitals or study authors, to assess the quality or strength of recommendations made?

**Methods**

We conducted a scoping review, following the preferred reporting items for systematic reviews and meta-analyses extension for scoping reviews guidance.[21].

***Sources and Searches***

Searches were performed on 28^th^ February 2019 and 30^th^ January 2021 using MEDLINE, EMBASE, PsychINFO and CINAHL. Search terms were iteratively developed to capture the key phases of incident investigation including terms for the incident, investigation and subsequent recommendations (appendix 1 for search terms, <http://links.lww.com/JPS/A565>). Searches were restricted to English language and studies published since 1999, when the Institute of Medicines’ seminal report, To Err is Human, was published,[22] prompting greater focus on patient safety.

***Study selection***

The aim of this review was to examine the routine investigation and recommendation generation processes that occur in hospitals.

Studies were included if they reported on a series of incidents occurring in hospital which were chosen for investigation by hospital-based staff, who also generated subsequent recommendations. Studies reporting on incidents from any clinical context or level of harm were included.

Studies were excluded if they reported data from:

1. Community, primary care, or primarily mental health care
2. Investigations/recommendations carried out or proposed outside of a hospital, for instance by an external research team or regional organisation.
3. Investigations primarily carried out for the purposes of research
4. Not published/peer-reviewed (e.g. conference papers)

Searches yielded 15,010 articles. The article title and abstracts were reviewed by WL. Random samples of 5% (n=720) were screened independently by both JOH and RL to check congruence. 246 articles were selected for full text review. Full text screening was undertaken by WL, with 10% independently screened by each of JOH and RL (n=20). Any discrepancies were discussed and resolved between authors. 11 articles met the inclusion/exclusion criteria (all agreed with WL, JOH and RL) and contributed to the review (Figure 1). Regular meetings with the other author (CV) allowed discussion of article eligibility.

***Data extraction and quality assessment***

The purpose of the review was to examine the nature of recommendations proposed within hospitals, which was not the primary aim of all the included studies, but those included did contain empirical data on recommendations.

We assessed study quality using the Quality Assessment for Diverse Studies (QuADS) tool.[23] This tool is a well cited approach to assessing the quality of methodologically heterogeneous studies, which demonstrates reliability and validity. [24,25] Following discussion of the application of the tool and relevance of quality scoring by all the authors, WL reviewed and scored all included articles. A random sample (n=4, 36%) of studies were independently reviewed and scored by JOH and RL, with disagreements resolved with discussion.

***Data Synthesis and analysis***

To address research questions one, two and four we undertook content analysis of the included studies using four stages; decontextualization, recontextualization, categorisation, and compilation.[26] Firstly authors read and made themselves familiar with the included studies before extracting ‘meaning units’ of text relevant to answering the aims of the review (decontextualization). After extraction of meaning units the remaining article text was checked for further relevant content (recontextualization). Next the extracted meaning units were split into specific areas relevant to each research question; the word count was reduced without losing the meaning/content (categorisation). The research questions were answered by condensing the extracted text, using the original study terms and language, as well as providing numerical counts of how often content was reported across the studies.

To address research question three we used the ‘action hierarchy’ (AH), proposed by the US Veteran Affairs National Center for Patient Safety to categorise the recommendations extracted from included studies.[16,27-30] Recommendations from the included studies were discussed by all the authors across two meetings and assigned to the core categories of the AH, then counted, in order to report frequency. If, following discussion, it was felt that a recommendation or category of recommendations did not fit into one of the AH categories, a new category was created and agreed.

**Results**

The characteristics of included studies (n=11) are summarised in Table 1. Included studies contained 4680 recommendations from 2818 investigations carried out across 171 hospitals.

***Country of origin***

Included studies were conducted in in the USA (n=4), UK (n=2), Australia (n=2), with one each from The Netherlands, Brazil, and Hong Kong.

***Clinical context and incident harm***

Studies reported data from across all clinical specialties (n=6), Pharmacy/Medication (n=1), Anaesthesia and intensive care (n=2), Paediatric care (n=2). Incidents reported within studies varied in their type (e.g. delay in care, fall, dispensing of medication) and resulting harm (see Table 1 for more detail).

***Quality Assessment***

The included studies demonstrated an average QuADS score of 56% (range 26-69%) Five of eleven studies lacked theoretical underpinning such as the discussion of an accident causation model. Half of the studies did not report, in sufficient detail, the justification of sampling or selection of data collection tools. Six studies had no evidence that research stakeholders had been involved in their planning or conduct. Four studies had limited or no discussion of their strengths or limitations. No studies were excluded based on quality.

***RQ1) Approaches to incident investigation used prior to the generation of recommendations***

~~Before an incident was investigated some studies reported that a hospital safety or quality team would judge what kind of investigation was needed and who should do it.[28,31-34]~~ Nine studies reported using Root Cause Analysis (RCA), [16,27-29,31,34-37] three used both RCA and the London Protocol, [16,34,36] and the remaining two, no specific tool or method.[32,33] Four studies reported a team of 2–8 staff (physicians, nurses and managers) undertook the investigation,[29,34-36] and two reported specific investigator training.[36,37] The remaining studies did not provide these details.

As part of the investigation process, three studies reported interviewing staff,[34-36] one of which specified that ‘incidents were reconstructed from a median of six interviews (n=3-15).[36] One study reported parents of children involved in incidents were interviewed ‘if felt to be useful’, and this occurred in 2/17 incidents.[36]

Four studies reported on the time spent undertaking investigations. This was highly variable, ranging from 3 to 90 hours.[27,34,36,37]. Three studies reported that investigations should be completed within a set period of time, ranging from 30 to 60 days,[29,31,35] although they did not specify if this was from when the incident occurred, was reported or the decision to investigate was made.

***RQ2) The processes for generating recommendations following a patient safety incident investigation***

None of the included studies reported using specific tools or methods for recommendation generation. One paper reported that staff and parents were invited to suggest recommendations, while none of the remainder reported this kind of stakeholder involvement.[36] Eight studies proposed that recommendations should prevent incident recurrence,[16,28,29,31,34-37] eliminate, mitigate or reduce a risk, hazard or ‘root causes’.[29,31,35,37] No purpose or aim for recommendations was stated in the remaining three studies.

***RQ3) The number and types of recommendations proposed***

A variety of terms were used to describe the recommendations generated following investigations. We present these terms in table 2, but as the terms were not clearly defined within the studies, we were not able to determine differences or similarities, and have therefore reported them as written. 4579 recommendations were extracted from 10 included studies (table 3), with an average of 3.7 (1-5) per investigation. Recommendations were not extracted from the eleventh included study due to insufficient detail to enable categorisation.[37] Six studies assigned recommendations to pre-determined categories based on i) the US Department of Veteran Affairs’ criteria, or ‘action hierarchy’,[16,27-29] ii) factors influencing clinical practice devised by Vincent et al,[36] or iii) the ‘hierarchy of intervention effectiveness’ (people versus system focused).[37]The remaining five studies developed their own categories based on analysis of their included recommendations.[31-35]

Education or training represented the most common recommendation (27.2%, n=1257), followed by new procedure/memorandum/policy (15%, n=676), change of process or routine (10.7%, n=500), and adjustment/improvement to policy or guideline (6.7%, n=306)). 14.1% of the extracted recommendations were too vague or unclear to categorise. Table 3 shows the full breakdown of recommendations by category. Recommendation categories 1-26, in table 3, are from the action hierarchy,[16,27-29] and categories 27-36 are those proposed by the study authors. 656 recommendations were categorised as ‘vague/unclear’ either by the authors of the included studies or authors of this review during analysis. Examples of ‘vague/unclear’ recommendations included ‘Medication incident action plan implemented’(n=3),[33] ‘policy, procedure and process actions’(n=5),[31] ‘provide counselling’ (n=280).[32]

**RQ4) Criteria used to assess the quality or strength of recommendations made**

Two, out of eleven, papers reported that the original internal hospital investigations made judgements of recommendation ‘quality’ or ‘strength’.[31,34] One study reported that the hospital prospectively tagged incidents to identify trends and therefore monitor for process improvements, though did not report any data in relation to this.[34] Another study reported that implemented actions (n=277) effectiveness was rated, by local managers, as “much better” (47.4%), “better” (37.0%), “same”(7.4%), “worse” (0%) or not reported or measured (8.2%).[31] While none the studies provided comprehensive data on incident recurrence, one study reported that similar incidents did reoccur despite multiple investigations.[35]

Included studies, in secondary analysis, used a range of terms or phrases to ‘judge’ recommendations as follows.

- **Effectiveness** (Hibbert 2018[16]; Kwok 2020[29]; Corwin 2017[31]; Figueiredo 2018[32]; Kellogg 2017[35]; Van der Starre 2014[36]; Robbins 2020[37])
- **Strength** (Hibbert 2018[16]; Morse 2012[27]; Hamilton 2019[28]; Kwok 2020[29]; Kellogg 2017[35])
- **Whether implemented** (Morse 2012[27]; Hamilton 2019[28]; Corwin 2017[31]; Kellogg 2017[35]; Van der Starre 2014[36])
- **Aimed at system level improvements or modifying processes** (Morse 2012[27]; Kwok 2020[29]; Kellogg 2017[35])
- **Likelihood they would prevent incident recurrence** (Morse 2012[27]; Kellogg 2017[35]; Van der Starre 2014[36])
- **Quality** (Morse 2012[27]; Robbins 2020[37])
- **Sustainability** (Hibbert 2018[16]; Kellogg 2017[35])
- **Efficacy** (Hamilton 2019[28])
- **Innovation** (Robbins 2020[37])

**Level of impact** (Morse 2012[27])

In eight out of eleven studies, authors discussed their approach to judging recommendations.[16,27-29,31,35-37]Four studies judged recommendations as strong, intermediate or weak based on the AH with some variations in the category descriptions and/or addition of further categories.[16,27-29] One study referenced a ‘Model of Sustainability and Effectiveness in RCA Solutions’,[35,38] while another reported effectiveness of recommendations according to the ‘Hierarchy of Intervention Effectiveness’ which proposes that ‘system-focused changes have greater impact’.[37,39] One paper commented on recommendations likelihood of preventing incident recurrence,[36] based on a classification of recommendation strength (Weak, Medium, Strong) proposed by the New South Wales Root Cause Analysis Review Committee.[40]

**Discussion**

To the author’s knowledge, this review represents the first review of the extant empirical evidence for the practice of generating recommendations in hospitals, specifically examining how and what recommendations were generated, as well as the way in which their effectiveness was judged. This process is central to the efforts to improve patient safety and healthcare quality globally. Our review highlights the paradoxical situation that, despite the ubiquity of recommendation generation, very little is known about it in practice. Our findings suggest that while RCA dominates as the approach to investigation, there are no specific tools or approaches used to generate recommendations. Recommendations focus on training or adding or improving policies. In other words, recommendations largely focus on staff knowledge and skills. There is a lack of agreement in the literature on how effectiveness of recommendations should be judged, meaning that there is very little understanding of what makes a ‘good’ recommendation. These findings raise some important issues, which we will address in turn.

**Recommendation generation is confused and unclear**

The variety of terms used to describe recommendations (table 2) and lack of consensus for categorisation suggests differences in vision and purpose at best, and confusion and disagreement at worst. While this review provides some steer in terms of the espoused investigation techniques, the actual process of how investigation outcomes result in specific recommendations remains opaque. We found that, beyond the investigators, there are committees or teams within hospitals as well as within local or regional organisations that review investigations and their findings; though what role these groups had in selecting or modifying recommendations is unclear. Studies in the wider literature have attempted to explore this process in practice. Braithwaite found a number of challenges to RCA such as time constraints, lack of resources and unwilling colleagues.[41] Another study suggested that recommendations may actually be related to other ongoing improvement work; i.e. the incident was used to support existing agendas rather than to generate new findings.[42] Further, an ethnography of investigations identified attempts by investigators to manage scrutiny and maintain reputations; and concluded that a failure to appreciate the complex organizational agendas as well as social and political influences on recommendation generation would likely hamper improvements in patient safety.[43] Beyond healthcare, studies of investigations from other domains, such as nuclear and rail, have demonstrated that the design of approaches to investigation and associated manuals lack emphasis or detail on the generation and evaluation of recommendations.[6] Another cross-domain study identified that there are a large number of cognitive, political contextual factors that influence the investigation and recommendation generation process, such as cost-benefit analysis, willingness of stakeholders to engage, or the experience or knowledge base of the investigator.[17] Collectively, these studies suggest that the generation of recommendations is likely to be a highly complex socio-political process with many stages and influences.[4,41-44] New approaches and tools for recommendation generation [45-48] are more likely to be successful if adapted and designed relative to the unique and complex context of healthcare.[49,50] Further research to understand the reality of the movement from investigation to recommendation generation is therefore important.

**Recommendations are classified as weak and lack system focus**

This review identified that less than 7% of the extracted recommendations might be considered ‘strong’ or system-focused, such as standardising equipment, architectural changes, or simplifying processes. Our findings provide further evidence for the continued tendency for ‘weaker’ recommendations that focus on improving individuals’ behaviour and practice, rather than the wider system deficiencies that contribute to incidents. This tendency, shown in numerous studies from across the globe,[5,51-58], suggests explanatory reasons beyond national culture or specific differences in healthcare systems, and is completely at odds with healthcare policy and safety research.[3,40,59] Further it would suggest, globally, healthcare organisations may have some way to go towards achieving a more just culture, with this focus on weaker individual-focussed recommendations both reflecting this and serving to reinforce it.[2]

RCA and frameworks, used to support investigation, have themselves been identified as narrowing the view of causation[4] or giving greater attention to causative factors relating to individuals.[60] With a tendency for investigations to identify individual factors,[60] it’s perhaps not surprising that recommendations are targeted at the same level. Others reasons for a lack of system-level recommendations include lack of investigator training, expertise,[5] or healthcare tailored guidance,[3] and difficulty in designing and implementing at the system-level.[15,49] This review highlights the continued predominance of RCA, despite the growing number of alternatives which might broaden investigations and identify a wider range of contributory factors and subsequent recommendations. [61-65]

**It is not clear how to judge recommendations**

Whilst the focus of recommendations at the weaker individual level has been widely challenged, a further compounding problem with recommendation generation is the lack of agreement on how to judge their effectiveness, and what makes a ‘good’ recommendation. The range of terms, in our included studies, such as ‘strength’, ‘quality’, ‘sustainability’ and ‘implementability’ indicate the complex nature of judging recommendations. Our review found two broad approaches: i) the use of pre-defined hierarchies of recommendation effectiveness; and, ii) assessing the effectiveness of recommendations over time.

Starting with hierarchies, this review demonstrates their widespread use but also variety and variation.[5,15,16,27,29,38-40,59,66-68] These hierarchies, largely originating from non-healthcare settings,[49,69] are used in healthcare with minimal empirical evidence.[38] They generally propose that recommendations targeted at the individual-level (e.g training and reminders) are weaker than those at the system-level (e.g. equipment design). Prior to this review, there have been challenges of the use of hierarchies to predict recommendation effectiveness,[48,49] with arguments that recommendations should be judged on how well they align with the identified risks and context,[47] their likelihood of effecting necessary change,[70] or level of system targeted for change.[48] Our review suggests that hierarchies may not yet be widely used in practice but with the growing number of variations, and lack of consensus, they have the potential to cause confusion for hospital safety teams looking to adopt evidence-based approaches. Beyond the need for empirical evaluation of these options, we suggest future research will also need to consider the practical application of these in healthcare.

The second approach to judging recommendation effectiveness appears to be ‘post-hoc’ measures, more specifically assessing what difference is made to processes and outcomes, as well as future incident occurence. In problem solving, determining the effectiveness of solutions is a key step.[69] There is a surprising absence of post-hoc measures reported within the included studies, with none of the included studies comprehensively reporting the rates of incidence recurrence. With ‘the prevention of incident recurrence’ being the most commonly quoted reason for incident investigation it is of note that these data are lacking within this review, as well as the wider literature.[4,5,44]

Beyond this review numerous studies have indicated that incident reporting systems (key for incident identification) only detect a minority of incidents that actually occur, and this number may be even lower for incidents resulting in harm.[71-73] Incident recurrence may be a poor marker of investigation success, if reporting remains unreliable. We contend that more research is needed to consider specifically what measures are appropriate for measuring recommendation or investigation effectiveness.

While Reasons organisational accident model is central to much of healthcare investigation practice,[3] the included studies demonstrate a lack of translation of the complexity and nuance of the original model. For instance, the recommendations largely focus on reducing error rates, rather than putting in place defences to more broadly improve system safety and quality or reduce the impact of an error if it does occur. The studies included within this review provide no evidence that carrying out investigations and generating recommendations improves the quality or safety of care. Further, there appears to be little consideration of the potential negative consequences of recommendations themselves.

**Limitations**

Despite the volume of incident reporting and investigation within healthcare there is a relative lack of peer-reviewed research with empirical data from ‘real-world’ hospital investigations. Relevant studies may have been excluded if there was ambiguity as to whether they reported data from usual practice within hospitals as this was the focus of the review. Due to the lack of studies exploring the specific aims of this review, the included study’s aims were not necessarily aligned with the aims of the review, rather relevant empirical data was extracted. Many of the included studies do not report the entire investigation process in detail, or the effect of recommendations which has impacted our ability to answer some of the review questions. It was not possible to analyse recommendations at level of the incident level, which would have allowed us to identify the proportion of recommendations at the individual and system levels. We recognise this would be an important area for future research. As we have focused on internal hospital investigations, as opposed to those at a regional or national level, there is a chance that this is one reason there are less observed recommendations targeting those contributory factors or organisations external to the hospital; internal hospital investigations may be more likely to focus on what they perceive they can change.[17] This review has focused on the generation of recommendations, but no assumption is made that ‘good’ recommendations will necessarily improve safety. Implementation of recommendations, and the challenges and barriers, is another important factor to consider but was beyond the scope of this review.

**Conclusions**

The aim of this review was to explore hospitals’ approaches to incident investigation, recommendation generation, the types of recommendations proposed and how their effectiveness is judged. While RCA dominates as the approach to investigation, how recommendations are selected remains unclear. Recommendations are generally classified as weak, focusing on improving individuals’ skills, knowledge and understanding so as to change behaviour rather than addressing deficiencies in the systems in which staff work. Our review demonstrates a lack of evidence and consensus regarding how recommendations should be judged for effectiveness. We argue that greater clarity is needed in terms of the purpose of investigations and the language used to describe them. Further, empirical work needs to explore and explicate how to generate appropriate recommendations, as well as how these approaches are adopted within the complex socio-technical context of healthcare.

Finally, we suggest that whilst incident investigations remain foundational to patient safety measurement and improvement, more enquiry is needed about their effectiveness or impact. The generation of recommendations themselves is only one step in the process. Both policy and practice will also need to engage with the growing body of literature and adopt a more evidenced-based approach to investigation and recommendation selection.

**References**

1. Macrae C. The problem with incident reporting. *BMJ Qual Saf* 2016;25:71–75.
2. Reason J. Human error: models and management. BMJ. 2000 Mar 18;320(7237):768-70. doi: 10.1136/bmj.320.7237.768. PMID: 10720363; PMCID: PMC1117770.
3. Woloshynowych M, Rogers S, Taylor-Adams S, et al. The investigation and analysis of critical incidents and adverse events in healthcare. *Health Technol Assess* 2005;9(19)
4. Peerally MF, Carr S, Waring J, et al. The Problem with root cause analysis. *BMJ Qual Saf* 2016;0:1-6
5. Card AJ, Ward J, Clarkson PJ. Successful risk assessment may not always lead to successful risk control: A systematic literature review of risk control after root cause analysis. *Journal of Healthcare Risk Management* 2012; 31(3)
6. Lundberg J, Rollenhagen C, Hollnagel E. What-You-Look-For-Is-What-You-Find – The consequences of underlying accident models in eight accident investigation manuals. Safety Science. Volume 47, Issue 10, December 2009, Pages 1297-1311
7. Wrigstad J, Bergström J, Gustafson P. Mind the gap between recommendation and implementation—principles and lessons in the aftermath of incident investigations: a semi-quantitative and qualitative study of factors leading to the successful implementation of recommendations. BMJ Open 2014;4:e005326. doi:10.1136/bmjopen-2014- 005326
8. HSIB (2022) How we improve patient safety Available at: https://www.hsib.org.uk/ (Accessed October 2022)
9. UKOM (2021) About Ukom Available at: https://ukom.no/om-ukom (Accessed: October 2022)
10. Kirkup B. The Report of the Morecambe Bay Investigation. 2015. Available at https://assets.publishing.service.gov.uk/government/uploads/system/uploads/attachment_data/file/408480/47487_MBI_Accessible_v0.1.pdf
11. Ockenden D. Ockenden Report: Findings, conclusions and essential actions from the independent review of the maternity services at the Shrewsbury and Telford Hospital NHS Trust. 2022. Available at: https://assets.publishing.service.gov.uk/government/uploads/system/uploads/attachment_data/file/1064302/Final-Ockenden-Report-web-accessible.pdf
12. Infected Blood Enquiry (2022) Available at: https://www.infectedbloodinquiry.org.uk/ Accessed November 2022
13. Latino RJ. How is the effectiveness of root cause analysis measured in healthcare? *Healthcare Risk Management* 2015; 35(2)
14. Dückers M, Faber M, Cruijsberg J, et al. Safety and risk management interventions in hospitals: A systematic review of the literature. *Med Care Res Rev* 2009;66(6 suppl):90S– 119S.
15. CDC NIOSH. NIOSH Topic: Engineering Controls. 2010. https://www.cdc.gov/niosh/topics/hierarchy/default.html. (accessed 1 Sep 2021)
16. Hibbert PD, Thomas MJW, Deakin A, et al. Are root cause analyses recommendations effective and sustainable? An observational study. *International Journal for Quality in Health Care*, 2018. 30(2), 124-131
17. Lundberg J, Rollenhagen C, Hollnagel E. What you find is not always what you fix-How other aspects than causes of accidents decide recommendations for remedial actions. Accident Analysis and Prevention, 2010 (42), 6, 2132-2139.
18. Carson-Stevens A, Hibbert P, Williams H, et al. Characterising the nature of primary care patient safety incident reports in the England and Wales National Reporting and Learning System: a mixed-methods agenda-setting study for general practice. *Health Serv Deliv Res* 2016;4(27).
19. Panagioti M, Kahn K, Keers RN, et al (2019) Prevalence, severity, and nature of preventable patient harm across medical care settings: systematic review and meta-analysis. *BMJ* 2019;366:14185
20. Gens-Barbera, Hernández-Vidal N, Vidal-Esteve E, et al. Analysis of Patient Safety Incidents in Primary Care Reported in an Electronic Registry Application. *International Journal of Environmental Research and Public Health*. 2021;18(17):8941
21. Tricco A, Lillie E, Zarin W et al. PRISMA Extension for Scoping Reviews (PRIMSA-ScR): checklist and explanation. Ann Intern Med. 2018;169:467-473
22. Kohn LT, Corrigan JM, Donaldson MS. To Err is Human: Building A Safer Health Care System. Washington DC: Institute of Medicine National Academy Press, 1999.
23. Harrison R, Jones B, Gardner P, et al. Quality assessment with diverse studies (QuADS): an appraisal tool for methodological and reporting quality in systematic reviews of mixed- or multi-method studies. *BMC Health Serv Res* 2021 15;21(1):144
24. Sirriyeh R, Lawton R, Gardner P, Armitage G. Reviewing studies with diverse designs: the development and evaluation of a new tool. J Eval Clin Pract. 2012;18(4):746–752.
25. Harrison R, Jones B, Gardner PH, Lawton RJ. Quality Assessment with Diverse Studies (QuADS): an appraisal tool for methodological and reporting quality in systematic reviews of mixed- or multi-method studies. BMC Health Serv Res. 2020.
26. Bengtsson M. How to plan and perform a qualitative study using content analysis. NursingPlus Open 2 (2016) 8-14
27. Morse RB, Pollack MM. Root Cause Analyses Performed in a Children’s Hospital: Events, Action Plan Strength, and Implementation Rates. *Journal for Healthcare Quality* 2012; 34(1):55-61
28. Hamilton MJ, McEniery JA, Osborne JM, et al. Implementation and strength of root cause analysis recommendations following serious adverse events involving paediatric patients in the Queensland public health system between 2012 and 2014. *Journal of Paediatrics and Child Health* 2019 55:1070-1076
29. Kwok YTA, Mah APY, Pang KMC. Our first review: an evaluation of effectiveness of root cause analysis recommendations in Hong King public hospitals. *BMC Health Services Research* 2020 20:507
30. National Patient Safety Foundation. RCA2: Improving Root Cause Analyses and Actions to Prevent Harm. 2015. https://psnet.ahrq.gov/issue/rca2-improving-root-cause-analyses-and-actions-prevent-harm (accessed 28 Mar 2022)
31. Corwin GS, Mills PD, Shanawani H, et al. Root Cause Analysis of ICU Adverse Events in the Veterans Health Administration. *The Joint Commission Journal on Quality and Patient Safety* 2017; 43:580-590
32. Figueiredo ML, Oliveira e Silva CS, Brito MFSF, et al. Analysis of incidents notified in a general hospital. *Rev Bras Enferm* 2018;71(1):111-9.
33. Irwin A, Ross J, Seaton J, et al. Retrospective analysis of DATIX dispensing error reports from Scottish NHS Hospitals. International Journal of Pharmacy Practice 2011 19:417-423
34. Zeng J, Nyflot MJ, Jordan LE, et al. Best practices for safety improvement through high volume institutional incident learning: lessons learned from 2 years. *J Radiat Oncol* 2016 5:323-333
35. Kellogg KM, Hettinger Z, Shah M, et al (2017) Our current approach to root cause analysis: is it contributing to our failure to improve patient safety? *BMJ Qual Saf* 2017;26:381-387
36. Van der Starre C, van Dijk M, van den Bos A, et al. Paediatric critical incident analysis: lessons learnt on analysis, recommendations and implementation. *Eur J Pediatr* 2014 173:1449-1457
37. Robbins T, Tipper S, King J, et al. Evaluation of Learning Teams Versus Root Cause Analysis for Incident investigation in a Large United Kingdom National Health Service Hospital. *J patient Saf* 2020 Mar 24
38. Hettinger AZ, Fairbanks RJ, Hegde S, et al. An evidence-based toolkit for the development of effective and sustainable root cause analysis system safety solutions. *J Healthc Risk Manag* 2013;33:11–20.
39. Cafazzo JA, St-Cyr O. From discovery to design: the evolution of human factors in healthcare. *Healthc Q* 2012;15
40. Taitz J, Genn K, Brooks V, et al. System-wide learning from root cause analysis: a report from the New South Wales Root Cause Analysis Review Committee. *Qual Saf Health Care* 2010 19(6):e63
41. Braithwaite J, Westbrook MT, Mallock NA, et al. Experiences of health professionals who conducted root cause analyses after undergoing a safety improvement program. *Qual Saf Health Care*. 2006;15(6):393-399
42. Tamuz M, Franchois K, Thomas E. What’s past is prologue: Organizational learning from a serious patient injury. *Safety Science* 2011;49:75-82
43. Nicolini D, Waring J, Mengis J. Policy and practice in the use of root cause analysis to investigate clinical adverse events: Mind the gap. *Social Science & Medicine.* 2011;73(2):217-225
44. Wu AW, Lipshutz AKM, Pronovost PJ. Effectiveness and Efficiency of Root Cause Analysis in Medicine. *JAMA* 2008;299(6)
45. Pham JC, Kim GR, Natterman JP, et al. ReCASTing the RCA: an improved model for performing root cause analyses. *Am J Med Qual* 2010;25:186–91.
46. Card AJ, Ward JR, Clarkson PJ. Generating Options for Active Risk Control (GO-ARC): introducing a novel technique. *J Healthc Qual* 2014;36:32–41.
47. Vincent C , Amalberti R. Strategies for safety. In: Vincent C, Amalberti R (eds). Safer Healthcare: Strategies for the Real World. Cham, Heidelberg, New York, Dordrecht, London: Springer 2016: 59–71.
48. Wood LJ, Weigmann DA. Beyond the corrective action hierarchy: A systems approach to organizational change. *International Journal for Quality in Health Care* 2020:438-444
49. Liberati EG, Peerally MF, Dixon-Woods M. Learning from high risk industries may not be straightforward: a qualitative study of the hierarchy of risk controls approach in healthcare. *International Journal for Quality in Health Care* 2018;30(1):39–43
50. Waterson P. Promoting systemic incident analysis in healthcare—key challenges and ways forwards, International Journal for Quality in Health Care 2021;33(4)
51. Mills PD, Neily J, Luan D, et al. Using Aggregate Root Cause Analysis to Reduce Falls and Related Injuries. *Joint Commission Journal on Quality and Patient Safety* 2005;31(1)
52. Mills PD, Neily J, Luan D, et al. Actions and Implementation Strategies to Reduce Suicidal Events in the Veterans Health Administration. *Joint Commission Journal on Quality and Patient Safety* 2006;32(3)
53. Mills PD, Neily J, Kinney LM, et al. Effective interventions and implementation strategies to reduce adverse drug events in the Veterans Affairs (VA) system. *Qual Saf Health Care* 2008;17:37-46
54. Mills PD, Huber SJ, Watts BV, et al. Systemic Vulnerabilities to Suicide among Veterans from the Iraq and Afghanistan Conflicts: Review of Case Reports from a National Veterans Affairs Database. *Suicide and Life-Threatening Behaviour* 2011;41(1)
55. Mills PD, Gallimore BI, Watts BV, et al. Suicide attempts and completions in Veterans Affairs nursing home care units and long-term care facilities: a review of root-cause analysis reports. *Int J Geriatr Psychiatry* 2016; 31:518–525
56. Charuluxananan S, Suraseranivongse S, Jantorn P, et al. Multicentered Study of Model of Anesthesia Related Adverse Events in Thailand by Incident Report (The Thai Anesthesia Incidents Monitoring Study): Results. *J Med Assoc Thai* 2008;91(7)
57. Hooker AB, Etman A, Westra M, et al. Aggregate analysis of sentinel events as a strategic tool in safety management can contribute to the improvement of healthcare safety. *International Journal for Quality in Health Care* 2019;31(2):110-116
58. Neily J, Silla ES, Sum-Ping SJT, et al. Anesthesia Adverse Events Voluntarily Reported in the Veterans Health Administration and Lessons Learned. *Anesth Analg* 2018;126(2):471-477
59. National Patient Safety Foundation. RCA2: Improving Root Cause Analyses and Actions to Prevent Harm. 2015. https://psnet.ahrq.gov/issue/rca2-improving-root-cause-analyses-and-actions-prevent-harm (accessed 28 Mar 2022)
60. Lawton R, McEachan RRC, Giles SJ*, et al* Development of an evidence-based framework of factors contributing to patient safety incidents in hospital settings: a systematic review *BMJ Quality & Safety*2012;21:369-380.
61. Igene OO, Johnson C. Analysis of medication dosing error related to Computerised Provider Order Entry system: A comparison of ECF, HFACS, STAMP and AcciMap approaches. Health Informatics Journal 2019,1-26
62. Isherwood P and Waterson P. To err is system; a comparison of methodologies for the investigation of adverse outcomes in healthcare. Journal of Patient Safety and Risk Management. 2021; 26(2), 64–73
63. Igene OO, Johnson CW, Long J (2021) An evaluation of the formalised AcciMap approach for accident analysis in healthcare. Cogn Tech Work 2022;24, 161–181
64. Patriarca R, Gravio GD, Woltjer R, et al. Framing the FRAM: A literature review on the functional resonance analysis method. Safety Science 2020; 129
65. Anjalee, J.A.L., Rutter, V. & Samaranayake, N.R. Application of failure mode and effects analysis (FMEA) to improve medication safety in the dispensing process – a study at a teaching hospital, Sri Lanka. BMC Public Health 21, 1430 (2021). https://doi.org/10.1186/s12889-021-11369-5
66. Hughes D. Root cause analysis: bridging the gap between ideas and execution. Topics in Patient Safety. 2006. https://www.patientsafety.va.gov/docs/TIPS/TIPS_NovDec06.pdf (accessed 16 Sep 2021)
67. Slade JJ, Wrzesniewski CE, Hunter OO, et al. Complementing Root Cause Analysis With Improvement Strategies to Optimize Venous Thromboembolism Prophylaxis in Patients With Epidural Catheters. *Quality Management in Health Care* 2020;29(4):253-259
68. VHA National Center for Patient Safety. Guide to Performing a Root Cause Analysis. 2021. https://www.patientsafety.va.gov/docs/RCA-Guidebook_02052021.pdf (accessed 28 Mar 2022)
69. Manuele FA. Achieving risk reduction, effectively. *Process Safety and Environmental Protection* 2006;84(B3):184-190
70. French SD , Green SE, O'Connor DA et al. . Developing theory-informed behaviour change interventions to implement evidence into practice: a systematic approach using the Theoretical Domains Framework. *Implement Sci* 2012;7:38.
71. Stanhope N, Crowley-Murphy M, Vincent C, et al. An evaluation of adverse incident reporting. *J Eval Clin Pract* 1999;5:5-12.
72. Nuckols TK, Bell DS, Liu H, et al. Rates and types of events reported to established incident reporting systems in two US hospitals. *Qual Saf Health Care* 2007;16(3):164-168
73. Sari A, Sheldon TA, Cracknell A, et al. Sensitivity of routine system for reporting safety incidents in an NHS hospital: retrospective patient case note review. *BMJ* 2007 334;79

**Figure 1 PRISMA-ScR flow diagram for study selection**

**Table 1 Included studies summary table**

**Table 2: Terms used to describe the recommendations following investigations**

**Table 3 Recommendations extracted from included studies**
